# Strawberry Tongue Associated With Epstein-Barr Virus Infection in a Seven-Year-Old Boy: A Case Report

**DOI:** 10.7759/cureus.94844

**Published:** 2025-10-18

**Authors:** Masazumi Miyahara, Kyoko Osaki

**Affiliations:** 1 Department of Pediatrics, Okanami General Hospital, Iga, JPN

**Keywords:** epstein–barr virus, mucocutaneous signs, pediatrics, rash, strawberry tongue

## Abstract

Strawberry tongue is classically associated with Kawasaki disease and scarlet fever, but it may also occur in other infectious or toxin-mediated conditions. We report a seven-year-old boy who presented with nasal obstruction, a generalized erythematous rash, and a strawberry tongue. He was afebrile and had no lymphadenopathy, hepatosplenomegaly, or pharyngeal exudate. Laboratory tests revealed mild liver enzyme elevation and a negative rapid antigen test for Group A *Streptococcus*. Serology confirmed primary Epstein-Barr virus (EBV) infection. No treatment was given, and all findings resolved spontaneously within 10 days of presentation. Although a causal relationship cannot be firmly established, careful exclusion of other etiologies supports EBV infection as the most plausible explanation for the development of strawberry tongue. This case highlights that EBV should be considered in the differential diagnosis of strawberry tongue when typical features of more common causes are absent.

## Introduction

Strawberry tongue serves as a visual diagnostic clue in pediatric patients presenting with mucocutaneous findings. While it is frequently described in well-characterized illnesses such as Kawasaki disease and scarlet fever [1,2], strawberry tongue is not pathognomonic; its presence alone does not establish a specific etiology and must therefore be interpreted in the clinical context. The characteristic appearance is thought to result from inflammatory hyperemia and desquamation of the lingual epithelium, accompanied by enlargement of fungiform papillae driven by cytokine-mediated mucosal inflammation. It may also occur in other infectious or toxin-mediated conditions, including *Yersinia pseudotuberculosis* (*Y. pseudotuberculosis*) infection and toxic shock syndrome [3,4], and drug reactions may also cause mucocutaneous changes; however, no clear association with strawberry tongue has been reported.

Epstein-Barr virus (EBV) infection most often presents with fever, pharyngitis, and lymphadenopathy, although atypical or mild clinical presentations are also recognized [5]. Mucocutaneous findings such as palatal petechiae, tonsillar exudates, and nonspecific rashes have been reported [5]; however, a systematic literature search in PubMed and Scopus using the terms “Epstein-Barr virus,” “strawberry tongue,” “lingual manifestations,” and “infectious mononucleosis” revealed no prior reports describing this association. Here, we report a case of primary EBV infection in a child who developed strawberry tongue and rash in the absence of fever or other systemic signs. This case underscores the importance of considering EBV in the differential diagnosis of strawberry tongue.

## Case presentation

A previously healthy seven-year-old Japanese boy presented with a three-week history of persistent nasal congestion and an eight-day history of a mildly pruritic, well-demarcated erythematous rash involving the face, trunk, and extremities. He had completed a short course of carbocisteine and cyproheptadine approximately ten days before presentation for upper respiratory symptoms, and no other medications or supplements had been taken. He had no fever, no recent travel, no known exposures to wild or domestic animals, and no history of insect bites.

**Figure 1:**
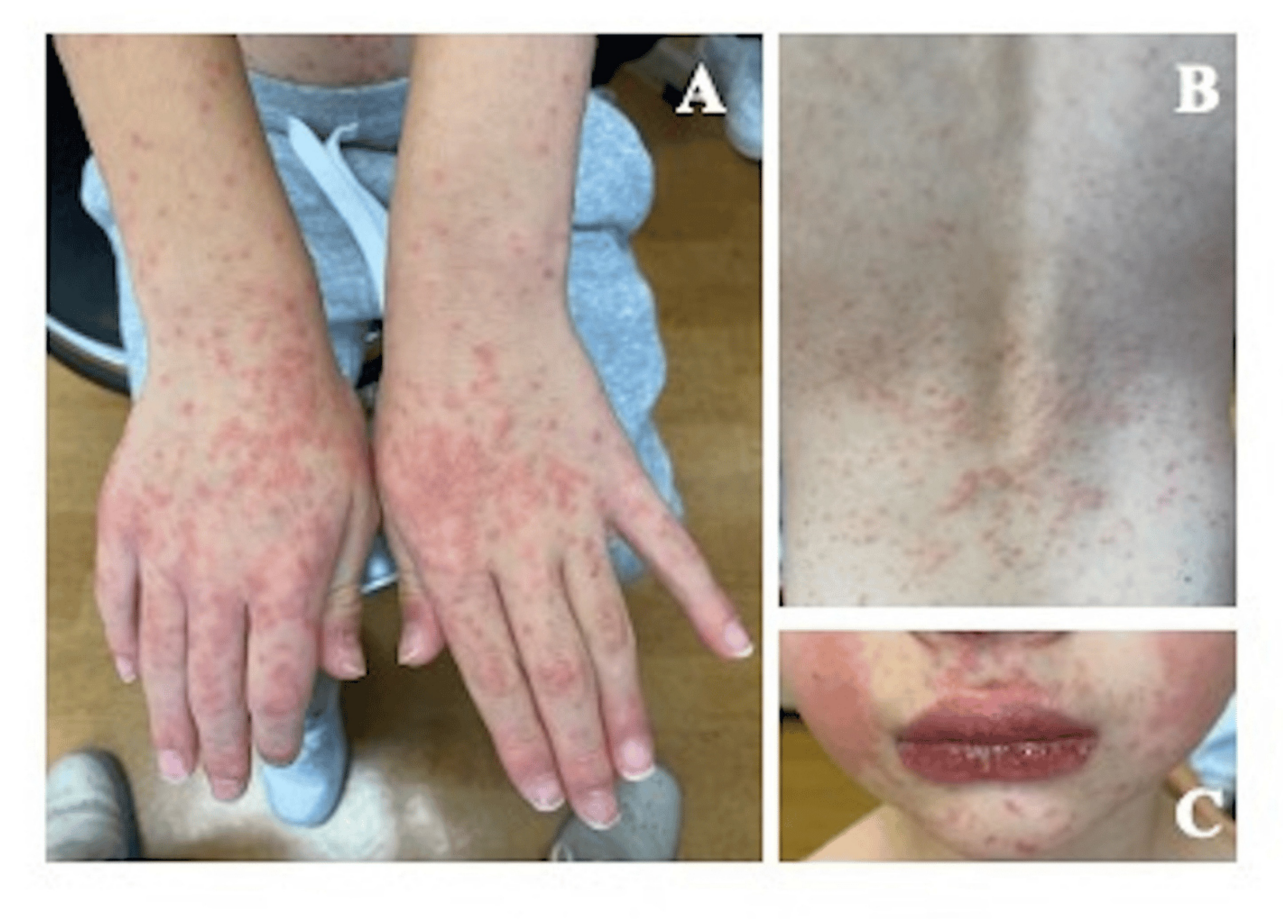
Cutaneous manifestations at initial presentation A: Erythematous maculopapular eruptions on the dorsal surfaces of both hands. B: Generalized erythematous eruptions involving the trunk. C: Erythematous facial rash.

**Figure 2:**
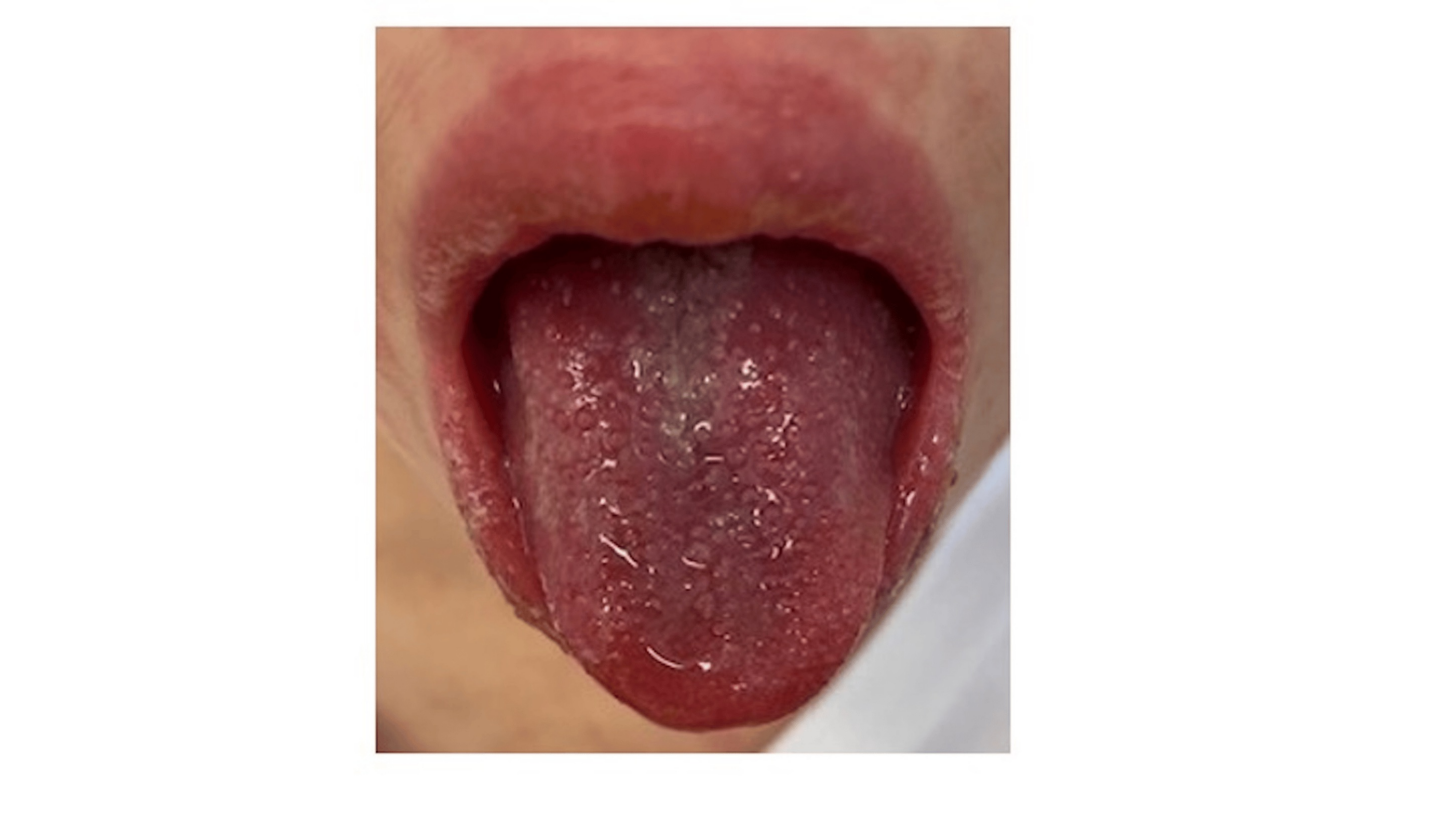
Strawberry tongue in a child with primary Epstein-Barr virus (EBV) infection Diffuse erythema of the tongue with prominent papillae, demonstrating the classic appearance of a “strawberry tongue” in a pediatric patient with primary EBV infection.

On examination, the patient appeared alert and well, with a body temperature of 36.9°C. A generalized maculopapular erythematous rash was noted on the face, trunk, and extremities (Figure 1). The tongue appeared diffusely erythematous with hypertrophic and prominent fungiform papillae, findings consistent with a “strawberry tongue” (Figure 2). This finding was first recognized at our initial examination, as it had not been noticed by the family. There was no conjunctival injection, cervical lymphadenopathy, hepatosplenomegaly, or peripheral extremity changes. No pharyngeal exudate was observed.

Laboratory testing revealed mild leukocytosis, with a white blood cell count of 12,400/μL (65% neutrophils, 29% lymphocytes, 2% monocytes, 4% eosinophils). C-reactive protein was mildly elevated at 0.31 mg/dL (reference <0.30), and liver enzymes were slightly elevated (aspartate aminotransferase 38 U/L, alanine aminotransferase 38 U/L; reference 10-35). Lactate dehydrogenase was 370 U/L (reference 110-225), suggesting mild hepatic involvement. A rapid antigen test for Group A *Streptococcus* (GAS) using a throat swab was negative. Serologic testing for EBV using enzyme immunoassay revealed positive viral capsid antigen (VCA)-IgM (5.7 index) and VCA-IgG (2.1 index), with negative Epstein-Barr nuclear antigen-IgG (0.3 index), consistent with primary EBV infection. Laboratory evaluation at presentation is summarized in Table 1. No specific treatment was administered. The rash and tongue changes resolved spontaneously within 10 days, and the patient remained well with no further symptoms thereafter.

**Table 1:** Laboratory findings at initial presentation ALT, alanine aminotransferase; AST, aspartate aminotransferase; BUN, blood urea nitrogen; Ch-E, cholinesterase; CPK, creatine phosphokinase; Crea, creatinine; CRP, C-reactive protein; EBNA-IgG, Epstein-Barr nuclear antigen-IgG; GAS, Group A *Streptococcus*; LAMP, loop-mediated isothermal amplification; LDH, lactate dehydrogenase; RBC, red blood cell count; T-Cho, total cholesterol; VCA-IgG, viral capsid antigen-IgG; VCA-IgM, viral capsid antigen-IgM; WBC, white blood cell count

Laboratory parameters	Patient value	Reference range
Peripheral blood test		
WBC	12400 /μL	4000-9000
Neutrophil	65%	39-81
Lymphocyte	29%	16-50
Monocyte	2%	2-10
Eosinophil	4%	2-10
RBC	511 × 10^4^/μL	400-520 × 10^4^
Hemoglobin	13.4 g/dL	13.0-17.0
Hematocrit	39.3%	38.0-49.0
Platelet	29.1 × 10^4^/μL	12.0-44.0 × 10^4^
Serum biochemical test		
Total protein	7.5 g/dL	6.5−8.５
Albumin	4.4 g/dL	4.1−5.３
Total bilirubin	0.33 mg/dL	0.2−1.2
Ch-E	381 U/L	214-466
AST	38 IU/L	10-35
ALT	36 IU/L	10-35
LDH	370 U/L	110-225
CPK	81 IU/L	50-200
T-Cho	95 IU/L	150-219
BUN	8.4 mg/dL	9.0-22.0
Crea	0.33 mg/dL	0.50-1.10
Sodium	140 mEq/L	138-145
Potassium	3.8 mEq/L	3.4-4.7
Chloride	104 mEq/L	99-108
CRP	0.31 mg/dL	0.00-0.30
Epstein-Barr virus serology		
VCA-IgG	2.1	<0.5
VCA-IgM	5.7	<0.5
EBNA-IgG	0.3	<0.5
Throat swab		
Rapid antigen test (GAS)	Negative	Negative
*Mycoplasma pneumoniae* antigen (LAMP)	Negative	Negative

## Discussion

When evaluating a child presenting with a strawberry tongue, the principal differential diagnoses include scarlet fever, Kawasaki disease, toxic shock syndrome, and *Y. pseudotuberculosis* infection [1-4]. Scarlet fever was considered unlikely in our patient. Although throat culture remains the diagnostic gold standard for GAS, this test was not performed. Nevertheless, several clinical and laboratory findings argued strongly against streptococcal infection. The patient was afebrile throughout the illness, exhibited no pharyngeal exudates or cervical lymphadenopathy, and presented with a generalized erythematous rash distinct from the typical scarlatiniform eruption of GAS, which is classically described as a fine, sandpaper-like rash often beginning on the trunk or lower abdomen [6]. In addition, the rapid antigen test for GAS was negative. Although spontaneous resolution alone does not definitively exclude streptococcal infection, the self-limited clinical course without antibiotic therapy, when considered together with these atypical features, makes this diagnosis highly improbable.

Kawasaki disease was also considered but excluded due to the absence of hallmark diagnostic criteria, including persistent fever, conjunctival injection, cervical lymphadenopathy, fissured lips, and extremity changes. Toxic shock syndrome, a toxin-mediated illness caused by *Staphylococcus aureus* or, less commonly, *Streptococcus pyogenes* [7], was excluded because the patient showed no evidence of systemic toxicity, desquamation, or hemodynamic instability. Although mild liver enzyme elevation was noted, there was no clinical evidence of significant multiorgan dysfunction. *Y. pseudotuberculosis* infection - a rare mimicker of Kawasaki disease [8] - was excluded on the basis of the absence of gastrointestinal symptoms, epidemiologic exposure, and systemic findings. Other conditions, such as Yellow fever and multisystem inflammatory syndrome in children (MIS-C) related to SARS-CoV-2 infection, were also considered in the differential diagnosis of strawberry tongue [9,10]. However, Yellow fever is not endemic in Japan, and the patient had no travel history to endemic areas, and MIS-C was unlikely, as this case occurred before the COVID-19 pandemic.

Strawberry tongue, defined by an erythematous dorsal tongue with preserved and hypertrophic fungiform papillae, is classically associated with toxin-mediated conditions such as scarlet fever and vasculitic syndromes like Kawasaki disease [11]. Although widely recognized as a hallmark feature of these conditions, it is not pathognomonic. Its appearance is attributed to cytokine-driven mucosal inflammation, a mechanism that may be shared across diverse infectious and inflammatory processes [11]. EBV infection is known to trigger systemic inflammatory responses through cytokine release, including interleukin-6 and tumor necrosis factor-alpha [12]. This immune activation may contribute to mucosal erythema and papillary hypertrophy, providing a plausible explanation for the development of strawberry tongue in this case.

Primary EBV infection typically presents with the triad of fever, pharyngitis, and lymphadenopathy [13]. However, atypical or mild presentations are recognized, and mucocutaneous manifestations such as palatal petechiae, tonsillar exudates, periorbital edema, and nonspecific rashes may also occur [13]. In this case, the absence of classic systemic features complicated the diagnosis, but positive serology, the presence of a generalized rash, nasal obstruction suggestive of tonsillar hypertrophy, and mild hepatic dysfunction, together with a self-limited clinical course, supported primary EBV infection as the underlying etiology. Although fever was absent, the cutaneous and mucosal manifestations strongly suggested that a substantial inflammatory process had occurred.

To date, strawberry tongue associated with EBV infection has not been reported in the literature. While a definitive causal relationship between EBV infection and the development of strawberry tongue cannot be established, alternative etiologies were carefully excluded, and EBV infection remains the most plausible explanation for the observed mucocutaneous findings.

A key limitation of this report is the inability to perform a throat culture for GAS, which remains the diagnostic gold standard for scarlet fever.

This case underscores the importance of considering EBV in the differential diagnosis of children presenting with strawberry tongue, particularly when features of more commonly associated illnesses are absent. Recognizing this atypical presentation may help avoid unnecessary investigations, misdiagnosis, or inappropriate treatment.

## Conclusions

We describe a child with primary EBV infection who developed a strawberry tongue and rash in the absence of fever or systemic features. Other common causes, including scarlet fever, Kawasaki disease, toxic shock syndrome, *Y. pseudotuberculosis* infection, Yellow fever, and MIS-C, were not supported by clinical or laboratory findings.

This case expands the known clinical spectrum of EBV-associated mucocutaneous manifestations and highlights the need to consider EBV infection in the differential diagnosis of strawberry tongue, particularly when typical features of more common causes are absent. Awareness of this atypical presentation may help clinicians avoid unnecessary investigations and inappropriate antibiotic use, promoting more accurate diagnosis and management in pediatric patients.
